# Deletion of the tyrosine phosphatase Shp2 in Sertoli cells causes infertility in mice

**DOI:** 10.1038/srep12982

**Published:** 2015-08-12

**Authors:** Xiaopeng Hu, Zhenzhou Tang, Yang Li, Wensheng Liu, Shuang Zhang, Bingyan Wang, Yingpu Tian, Yinan Zhao, Hao Ran, Wenjie Liu, Gen-Sheng Feng, Jianwei Shuai, Haibin Wang, Zhongxian Lu

**Affiliations:** 1 School of Physics and Mechanical & Electrical Engineering, School of Pharmaceutical Sciences, Xiamen University, Xiamen, Fujian 361005, China; 2 State Key Laboratory of Cellular Stress Biology, School of Pharmaceutical Sciences, Xiamen University, Xiamen, Fujian 361005, China; 3 State Key Laboratory of Reproductive Biology, Institute of Zoology, Chinese Academy of Sciences, Beijing 100101, China; 4 Department of Pathology, and Division of Biological Sciences, University of California San Diego, La Jolla, CA 92093, USA

## Abstract

The male’s ability to reproduce is completely dependent on Sertoli cells. However, the mechanisms governing the functional integrity of Sertoli cells have remained largely unexplored. Here, we demonstrate that deletion of Shp2 in Sertoli cells results in infertility in mice. In Shp2 knockout mice (SCSKO), a normal population of Sertoli cells was observed, but the blood-testis barrier (BTB) was not formed. Shp2 ablation initiated the untimely and excessive differentiation of spermatogonial stem cells (SSCs) by disturbing the expression of paracrine factors. As a consequence, the process of spermatogenesis was disrupted, and the germ cells were depleted. Furthermore, Shp2 deletion impaired the cell junctions of the primary Sertoli cells and failed to support the clonal formation of SSCs co-cultured with SCSKO Sertoli cells. As expected, Shp2 restoration largely restores the cell junctions of the primary Sertoli cells and the clonal formation of SSCs. To identify the underlying mechanism, we further demonstrated that the absence of Shp2 suppressed Erk phosphorylation, and thus, the expression of follicle-stimulating hormone (FSH)- and testosterone-induced target genes. These results collectively suggest that Shp2 is a critical signaling protein that is required to maintain Sertoli cell function and could serve as a novel target for male infertility therapies.

Sertoli cells (SCs) play a critical role in the physiology and pathology of the testes in mammals. In the embryo, SCs are the first somatic cells to differentiate in the testes and are thought to direct further testes development^1^,^2^,^3^. At puberty (approximately 14 days old in mice), SCs enter into the differentiation process, which includes a cessation of proliferation, alterations in protein expression and transcription, and functional maturation^4^,^5^. Mature SCs create the blood-testis barrier (BTB) to provide microenvironments for spermatogenesis and secrete many functional products to nourish germ cells and organize the events of spermatogenesis^2^,^3^,^6^. In particular, SCs produce numerous factors (such as glial cell line-derived neurotrophic factor (GDNF), stem cell factor (SCF), fibroblast growth factor 2 (FGF2), bone morphogenic protein 4 (BMP4)) to initiate the differentiation of spermatogonial stem cells (SSCs) and maintain the balance between SSC self-renewal and differentiation^7^,^8^,^9^,^10^. Thus, any abnormalities in the population and function of SCs result in aberrant spermatogenesis and eventually infertility^1^,^2^.

SCs are a central target for the regulation of spermatogenesis^1^,^2^. In mammals, spermatogenesis employs an elaborate regulatory mechanism, which is controlled by a multitude of regulators, including hormones (such as FSH, androgen)^1^,^11^, growth factors (transforming growth factor beta (TGF-β), tumor necrosis factor alpha (TNFα), and GDNF) endotoxins, and proinflammatory cytokines^1^,^3^,^12^,^13^. Based on the structure of the testes, these extracellular regulators primarily target SCs and generate a complex network of intracellular signaling pathways (including protein kinase A and C (PKA/PKC), calcium/calmodulin, mitogen-activated protein kinase (MAPK) and phosphoinositide 3-kinase (PI3K)/Akt pathways)^12^,^14^,^15^. In particular the FSH receptor is exclusively expressed on SCs, not germ cells; thus, FSH signaling is mediated through SCs^11^. The intracellular signaling pathways in SCs were integrated to produce the terminal biological effects on spermatogenesis^1^,^2^,^12^. For example, testosterone together with TNFα and TGF-β promotes the junction integrity of the BTB^16^. FSH and testosterone activate the MAPK pathway to stimulate SC proliferation^16^,^17^. However, little information is known about how these signaling pathways are coordinated and integrated in SCs.

FSH and testosterone trigger classical and non-classical cytoplasmic signal transduction pathways^16^,^18^,^19^. The latter typically contributes to the crosstalk of signaling activated by growth factors and cytokines^12^,^19^,^20^. Receptor-associated proteins (such as PI3K, c-Src, focal adhesion kinase (FAK) and c-Yes) may play important roles in the coordination of intracellular signaling pathways in SCs^1^,^19^,^21^. The non-receptor tyrosine phosphatase Shp2 typically mediates cytokine signal transduction as a receptor-associated protein^22^,^23^. Shp2 negatively regulates several tyrosine kinase receptor signaling pathways, such as insulin, leptin, inflammatory cytokines, via its tyrosine phosphatase domain^22^,^23^. However, Shp2 also positively enhances several signaling pathways (epidermal growth factor (EGF), insulin, platelet-derived growth factor (PDGF)) by triggering Ras-Erk and PI3K/AKT cascades^22^,^23^,^24^. Based on its dual regulation in cytoplasmic signaling pathways, Shp2 regulates cell proliferation, differentiation, migration and apoptosis and plays crucial roles in organ development (e.g., heart, breast and fat), immunology, metabolism and carcinogenesis^12^,^22^,^25^,^26^,^27^,^28^. Recently, Shp2 was demonstrated to mediate estrogen signaling by interacting with the extranuclear estrogen receptor (ER) in breast cancer cells^29^, indicating that Shp2 may play a role in the crosstalk between hormones and cytokines in SCs.

Shp2 is expressed in germ cells, Leydig cells and SCs in mice testes^30^. Patients with Noonan syndrome (Shp2 gene *ptpn11* mutation) exhibit a hypospermatogenesis phenotype with reduced seminiferous tubules and immature SCs^31^. The overexpression of a constitutively active Shp2 mutant, Shp2Q79R, in primary rat SCs upregulates Erk1/2 activation and disorganizes the BTB structure^32^. These data indicated that Shp2 is involved in regulating signaling in SCs, but the physiological role of Shp2 in spermatogenesis is not fully understood. In this study, we conditionally deleted the Shp2 gene in SCs using two transgenic mouse models and found that Shp2 deficiency caused infertility, excessive differentiation of SSCs and an abnormal BTB in mice. As a result, spermatogenesis was disrupted, and the germ cells were exhausted due to excessive SSC differentiation. Our studies discovered a novel role for Shp2 in mediating effects on SCs during spermatogenesis.

## Results

### Conditional deletion of Shp2 in Sertoli cells results in infertility in mice

**Figure 1:**
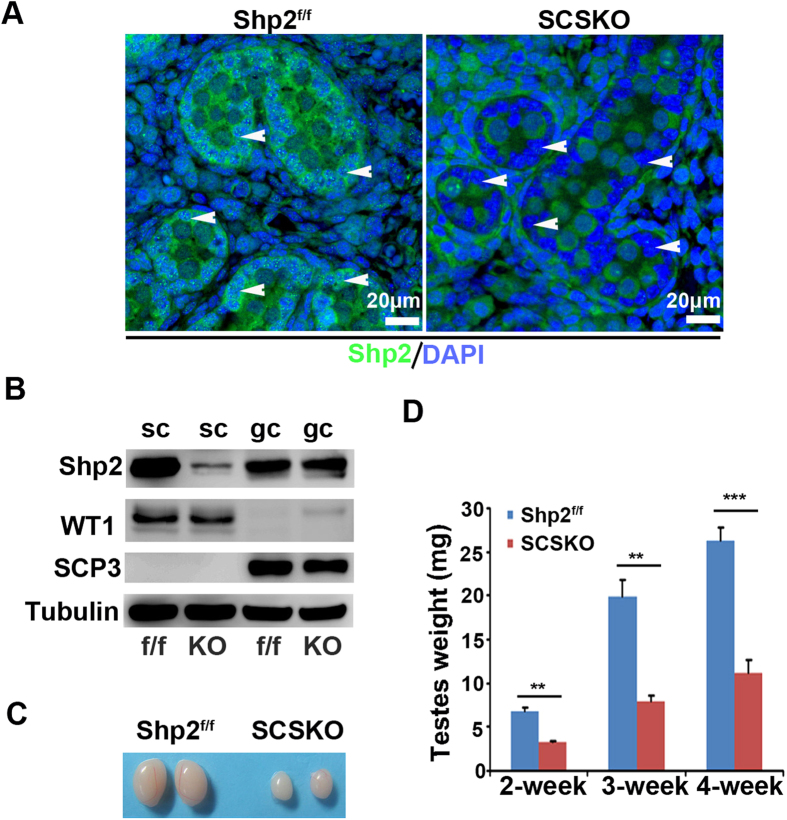
Shp2 depletion in Sertoli cells impeded testes development in mice. (**A**) Shp2 expression was detected with immunofluorescence staining in the seminiferous tubules at embryonic day 18 (E18). Arrowheads indicated Sertoli cells. The cell nucleus was stained with DAPI. (**B**) Shp2 protein levels were analyzed by Western blotting in primary Sertoli cells and germ cells isolated from 14-day-old mice. Wt1 is a marker of SCs and SCP3 is a marker protein of GCs. Tubulin was used as a loading control. The full-length blots are presented in Supplemental Figure S6. (**C**) The stereomicroscopy images of the testes from Shp2f/f and SCSKO male mice at 4 weeks. (**D**) The average weight of the testes from 2-, 3- and 4-week-old Shp2^f/f^ and SCSKO mice. The values are expressed as the mean ± standard error of the mean (SEM) from 8 mice. Statistical analysis was performed using Student’s *t*-test. Asterisks denote the statistical significance, **P < 0.01; ***P < 0.001.

To address the role of Shp2 in SCs and male reproduction, we generated Shp2 conditional knockout mice using the loxp-cre system. Shp2^loxp/loxp^ mice (Shp2^f/f^) were mated with Amh-Cre mice, which expressed the Cre recombinase in SCs at embryonic 14 day (E14)^33^. Immunofluorescence staining revealed that the Shp2 protein was specifically deleted in SCs in the Shp2^f/f^/Amh-Cre mice (SCSKO) at embryonic day 18 (E18) (Fig. 1A, Shp2 protein was labeled in green, and the arrowheads indicated SCs). Additionally, Shp2 protein was clearly absent in Western blots of primary SCs, but not germ cells isolated from 14-day-old mice (Fig. 1B). As a control, the protein level of Wilms tumor 1 (Wt1) was not altered. Then, we evaluated the impact of Shp2 deletion in SCs on male fertility using a successive breeding assay (see details in the Methods section). Adult SCSKO or littermate control male mice were mated with wild type female mice. The probability of observing a vaginal plug was not different between the two groups of mice (unpublished data). After a total of 40 matings, Shp2^f/f^ mice sired a total 271 pups (37 litters), whereas the SCSKO male mice did not produce any progeny (Table 1). As a confirmation, no mature sperms were found in epididymides of the SCSKO male mice (Supplemental Fig. 1). In addition, although the SCSKO mice had a normal body weight, the average weight of their testes was obviously reduced compared with littermate controls at different ages (Fig. 1C,D). Furthermore, Shp2 was also deleted in both SCs and Leydig cells using an inhibit α-Cre model (Supplemental Fig. 2A)^34^. The male knockout mice were also infertile, and the testes were small and exhibited a damaged structure (Supplemental Fig. 2B and C). These results indicated that Shp2 deficiency in SCs could injure testes development and induce male infertility in mice.

### The architecture of the seminiferous tubules is damaged and the germ cells are gradually lost in SCSKO mouse testes

**Figure 2:**
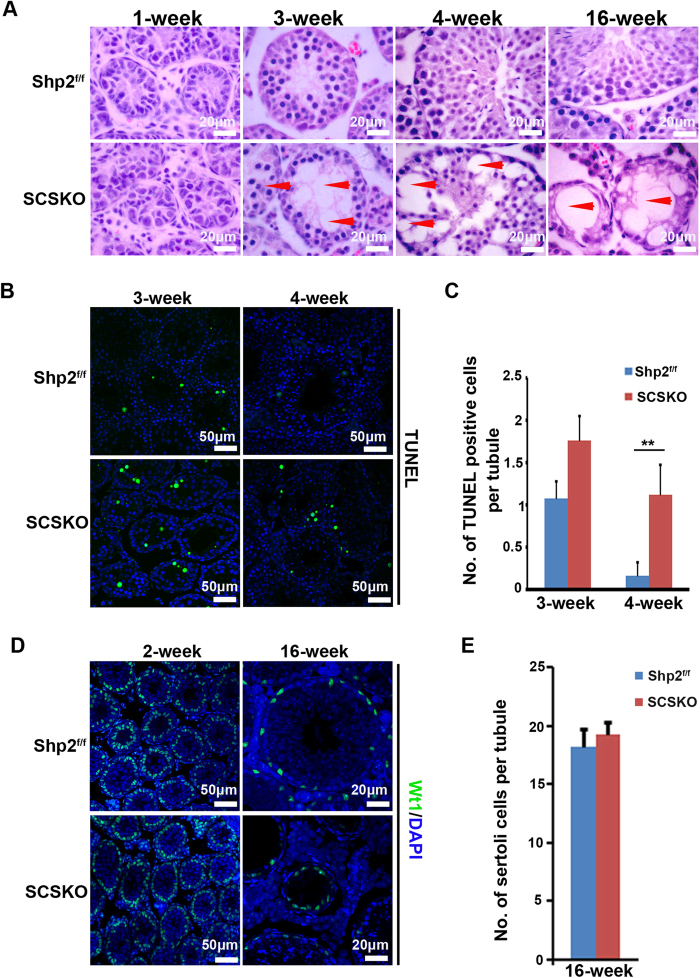
Shp2 ablation caused abnormal seminiferous epithelium and the gradual loss of the germ cells. (**A**) The histological structure of the seminiferous epithelium stained with hematoxylin and eosin (H/E) in Shp2^f/f^ and SCSKO mice at 1, 3, 4 and 16 weeks. Arrowheads indicated the abnormal phenotypes of the seminiferous epithelium. (**B**) Apoptotic cells in the seminiferous epithelium were detected by TUNEL staining. (**C**) The quantification of the number of apoptotic cells per tubule in the intersecting surface of the testes based on TUNEL analysis. (**D**) Sertoli cells labeled by Wt1 immunofluorescence staining in the seminiferous epithelium. (**E**) The quantification of the number of Sertoli cells per tubule in the intersecting surface of the testes. The cell nucleus was stained with DAPI. The values are expressed as the mean ± SEM from at least 5 mice from different litters. Statistical analysis was performed using Student’s *t*-test. Asterisks denote the statistical significance; **P < 0.01.

To investigate the testes defects in SCSKO mice, a histological analysis of the testes was performed by hematoxylin and eosin (H/E) staining (Fig. 2A). No obvious difference in the testicular structure is noted between one-week-old Shp2^f/f^ and SCSKO mice (Fig. 2A, left images). However, at 3 weeks old, various abnormalities appeared in the seminiferous tubules of the SCSKO mice. The number of cells decreased, and the cells exhibited a disordered arrangement (Fig. 2A, left, second image on the bottom panel, arrowhead indicated). At 4 weeks old, the seminiferous tubules in the Shp2^f/f^ mice contained typical spermatogenic waves and elongated sperm (Fig. 2A, left, third image on the top panel), whereas most of the tubules (84%) in the SCSKO mice were obviously damaged and exhibited cell loss, vacuolization, a chaotic arrangement of germ cells, and the absence of elongated sperm (Fig. 2A, right, second image on the bottom panel, arrowhead indicated). At 16 weeks old, the control testes exhibited fully developed tubules, which had an enlarged cavity and contained multiple types of germ cells (Fig. 2A, right top image). In contrast, the seminiferous tubules in the SCSKO mice were heavily destroyed. Some tubules (approximately 31%) lost all of the germ cells, became significantly thinner and contained only SCs (Fig. 2A, right bottom image, arrowhead), which is similar to a SC-only phenotype. In addition, interstitial Leydig cell hyperplasia was observed in the SCSKO mice.

Then, cellular apoptosis in the testes was analyzed with terminal deoxynucleotidyl transferase-mediated dUTP nick-end labeling (TUNEL) staining (positive cells are green). The apoptotic cells in the testes of three-week-old SCSKO mice were moderately increased (Fig. 2B), but the increase did not reach significance (Fig. 2C) compared with Shp2^f/f^ mice. However, the testes of knockout mice contained more apoptotic cells than the control testes at 4 weeks old (Fig. 2B,C). Furthermore, we measured the population of SCs using Wt1 as a tag (positive cells are green)^35^ and found that the number of SCs in the SCSKO mice did not differ from the control mice (Fig. 2D,E). These results suggested that Shp2 deletion in SCs at an early stage of development had no influence on the SC population but may disturb the functional maturation of SCs.

### Shp2 is required for the functional blood-testis barrier

**Figure 3:**
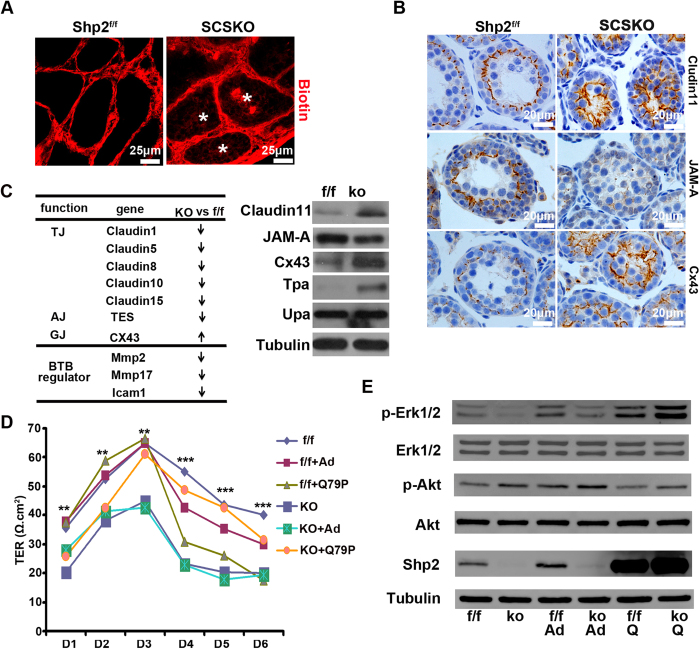
BTB integrity was disrupted by Shp2 deletion. (**A**) BTB permeability was assayed with the biotin tracer (red) using confocal microscopy in two-week-old mice. Asterisk indicates the biotin tracer in the adluminal compartment of the seminiferous tubules. (**B**) Immunohistochemical staining of the BTB junction proteins Cx43, Claudin11 and JAMA in seminiferous tubules in two-week-old mice. (**C**) Altered BTB-related genes in the microarray gene database, and the protein levels of various genes were analyzed by Western blotting in testes from two-week-old mice. The full-length blots are presented in Supplemental Figure S7. (**D**) The quantity of the cell junctions between primary Sertoli cells isolated from two-week-old mice was measured by transepithelial resistance (TER) assays. The experiments were repeated at least three times, and one representative result was shown. (**E**) Shp2 expression and Erk and Akt phosphorylation in primary Sertoli cells used in the TER assays. Tubulin was used as a loading control. The full-length blots are presented in Supplemental Figure S8. Ad, adenovirus virus; f/f, Shp2^f/f^ cells; ko, SCSKO cells; Q79P, constitutively active Shp2 mutant. The values are expressed as the mean ± SEM. Statistical analysis was performed using Student’s *t*-test. Asterisks denote the statistical significance, **P < 0.01; ***P < 0.001.

Mature SCs first created the BTB to support spermatogenesis^1^,^2^. BTB integrity was evaluated by using a biotin tracer to determine whether assembly of the BTB was impaired by Shp2 deletion^36^. The biotin tracer solution was injected into the interstitium of the testes in 2-week-old mice. After 2 hours, the animals were euthanized, and the biotin signals in testis sections were detected using a confocal microscopy. In the Shp2^f/f^ male mice, the biotin tracers were restricted to the interstitial space and the basal compartment (Fig. 3A, red color in the left image), whereas the biotin tracer penetrated into the adluminal compartment in most of seminiferous tubules (77%) in the SCSKO males (Fig. 3A, asterisk indicates the red color in the right image). These observations suggested that the BTB permeability was increased and the formation of the BTB was disturbed in SCSKO mice.

Then, we examined the expression and location of various junction proteins essential for normal BTB assembly, including connexin 43 (Cx43)^37^, claudin11^38^ and junctional adhesion molecule A (JAMA)^39^. In normal mice, the BTB was formed at approximately 2 weeks old, and the junction proteins Cx43, claudin11 and JAM-A were arranged as a regular cycle, which divided the seminiferous tubules into two parts (basal and adluminal compartment) (Fig. 3B, left images). However, these junction proteins displayed aberrant localization and were dispersed over the entire tubules in SCSKO mice. In addition, claudin11 and Cx43 expression was significantly increased, but JAMA expression was significantly reduced (Fig. 3B, right images). Together, these observations suggested that Shp2 deletion inhibited the original assembly of the BTB.

Because the number of SCs was not affected by Shp2 deficiency, the disarranged BTB may result from dysfunctional SCs. To fully understand the effect of Shp2 deletion on SC function, we screened gene expression profiles in the mouse testes at embryonic day 16.5 (E16.5), postnatal day 3 (D3), 1 week (1W) and 2 weeks (2W) (the data have been submitted to GEO, GSE68829) (Supplemental Fig. 3). The expression of approximately four hundred genes was altered by Shp2 ablation in SCs at 14 days old when the BTB was initially formed. In these altered genes, the genes from signaling pathways for cell adhesion, focal adhesions, tight junctions and gap junctions were enriched (Supplemental Fig. 4), including junction genes (Cx43, JAMA, claudin11, claudin1, claudin5) and BTB regulators (matrix metalloproteinase 2 (MMP2), MMP17, intercellular adhesion molecule 1 (ICAM1) and tissue-type plasminogen activator (TPA))^40^,^41^,^42^ (Fig. 3C, left table). Using real-time PCR and Western blotting, we confirmed the expression of some of these genes (Fig. 3C, right panel; Supplemental Fig. 5). The integrity of the BTB primarily depended on the proper assembly of tight junctions, gap junctions and adherent junctions between SCs or between SCs and germ cells. These data suggested that Shp2 deletion blocked the normal BTB assembly by disturbing the expression and location of junction proteins in SCs.

To further investigate the effects of Shp2 on BTB formation, we isolated and cultured primary SCs from mice *in vitro* and then assessed the integrity of the cell junctions using transepithelial resistance (TER) assays^43^. The electrical resistance value of the SC monolayer is increased when the cells form integrated tight junctions. In normal SCs, the electrical resistances increased with cell growth and reached a peak level on the third day (Fig. 3D, f/f and f/f+ad lines). However, the electrical resistances of the SCSKO SCs maintained a low level and did not reach the peak level during cell growth (Fig. 3D, KO and KO+ad lines). As expected, when the Shp2 protein was restored in SCSKO SCs by adenovirus infection with a constitutively active Shp2 mutant (Shp2/Q79P), the tight junction was rescued, and the electrical resistances also peaked on the third day (Fig. 3D, KO+Q79P lines). These observations indicate that the lack of Shp2 impaired the tight connections of SCs. In addition, Shp2 is a necessary protein in the Sertoli cell junctions. To our surprise, Shp2 hyperactivity also impaired cellular connections, and the resistance value decreased when wild type SCs were infected with adenovirus containing Shp2-Q79P (Fig. 3D, f/f+Q79P lines). In addition, we analyzed the cytoplasmic signaling in SCs with or without Shp2 and found that Shp2 ablation decreased Erk phosphorylation (p-Erk) (Fig. 3E, the left second and fourth band at top first panel), but increased Akt activity (p–Akt) (Fig. 3E, the left second and fourth band at top third panel). Shp2 restoration in SCSKO SCs also recovered the level of p-Erk and reduced Akt activity (Fig. 3. E, right first band at top first or third panel). These results suggested that Shp2 governed the assembly of cell junctions by coordinating the cytoplasmic signaling pathways.

### Shp2 deletion in Sertoli cells initiates and enhances untimely SSC differentiation

**Figure 4:**
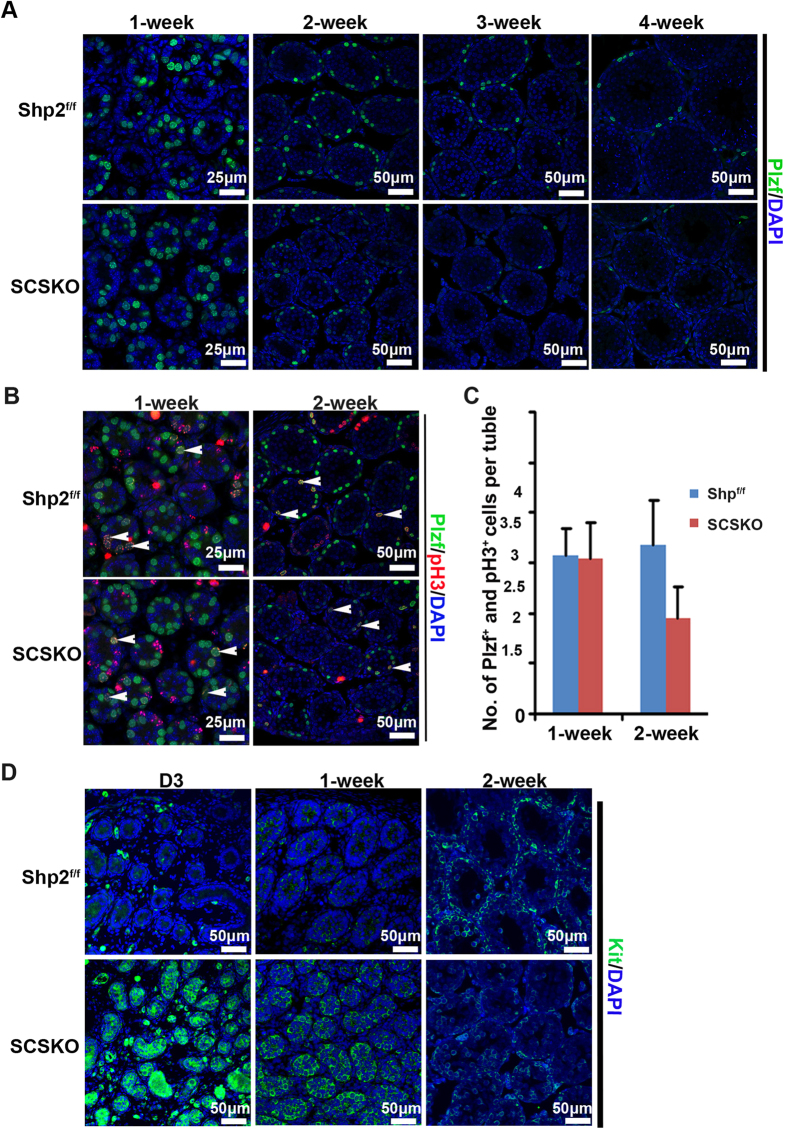
Shp2 deficiency decreased the number of SSCs and initiated the untimely and excessive differentiation of SSCs in testes. (**A**) Undifferentiated SSCs in the seminiferous tubules were identified by PLZF-positive immunofluorescence staining (green). (**B**) Proliferating SSCs in the seminiferous tubules were identified with PLZF and PH3-double positive immunofluorescence staining (yellow). Asterisks indicated positive SSCs (yellow). (**C**) Quantification of the proliferating SSCs (PLZF and Ph3 double stained cells) per tubule in the intersecting surface. The values are expressed as the mean ± SEM from at least 5 mice from different litters. Statistical analysis was performed using Student’s *t*-test. Asterisks denote statistical significance. **P < 0.01. (**D**) The differentiated SSCs in the seminiferous tubules were identified by c-Kit-positive immunofluorescence staining (green). The cell nucleus was stained with DAPI. The experiments were repeated at least three times, and one representative result is presented.

Another major function of mature SCs is to appropriately initiate the differentiation of SSCs and maintain the balance between SSC self-renewal and differentiation. Any abnormalities in these processes impair SSC maintenance and spermatogenesis^1^,^2^. In adult SCSKO mice, the germ cells were depleted in many tubules, indicating that SSC maintenance may have been disrupted by Shp2 deletion in SCs. To address this question, we analyzed the proliferation and differentiation of SSCs using immunofluorescence staining with a specific biomarker. Promyelocytic leukemia zinc finger (PLZF), a marker of SSCs and undifferentiated spermatogonia^23^,^44^, was stained in testis sections from postnatal week 1 to 4 (Fig. 4A). In normal mice, the PLZF-positive germ cells were maintained at high levels at 1 to 2 weeks of age (Fig. 4A, left two images in the top panel), but were reduced as the mice grew (Fig. 4A, right two images in the top panel). The number of PLZF-positive germ cells in SCSKO mice was similar to that in Shp^f/f^ males only at 1 week of age (Fig. 4A, left first image in the bottom panel), but these numbers rapidly decreased at older ages (Fig. 4A, right three images in the bottom panel). These observations clearly demonstrate that SSC maintenance was impaired.

**Figure 5:**
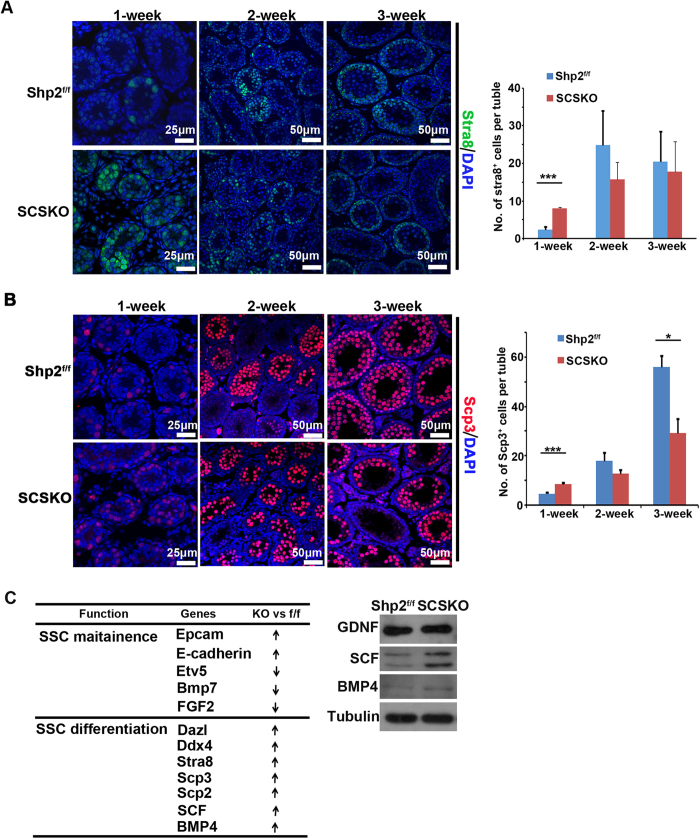
Shp2 deletion enhanced spermatogonia differentiation and altered the expression of proteins essential for SSC maintenance. (**A**) The differentiated spermatogonia in the seminiferous tubules are denoted by stra8-positive immunofluorescence staining (green). (**B**) The spermatocytes were identified as Scp3 positive (red). Asterisks denote statistical significance. The cell nucleus was stained with DAPI. Tubulin was used as a loading control. The experiments were replicated at least three times, and one representative result is presented. *P < 0.05: **P < 0.01; ***P < 0.001. (**C**) Altered genes related to SSC maintenance in the microarray gene database, and the protein level of some of these genes was analyzed by Western blotting in primary Sertoli cells at postnatal day 10. The full-length blots are presented in Supplemental Figure S9.

Then, cells were stained for a cell proliferation marker protein, PH3^13^. Although the PLZF and PH3-double labeled germ cells in SCSKO mice exhibited a small decrease, they did not reach a statistically significant difference compared with the normal mice at 2 weeks old (Fig. 4B,C, asterisk). Then, germ cell differentiation was evaluated by c-Kit staining. c-Kit is expressed from late aligned spermatogonia (A_al_) onward and promotes the transition of A_al_ into A1 spermatogonia, which is thought to be an initial step in spermatogenesis^45^. In normal mice, c-Kit was originally expressed in mice at approximately 6 days after birth and reached a high level at 2 weeks old (Fig. 4D, top panel). At that time, the c-Kit-positive spermatogonia were limited near the basal compartment by the BTB. However, in the SCSKO mice, strong Kit signals were observed at 3 days and 1 week old (Fig. 4D, left first and second image in the bottom panel). At two weeks of age, the c-Kit signals in the SCSKO mice were degraded (Fig. 4D, right image in the bottom panel). These data revealed that Shp2 deletion in SCs initiated an untimely and excessive differentiation of SSCs. Furthermore, cells were stained for stimulated by retinoic acid gene 8 (STRA8) and (synaptonemal complex protein 3) SCP3, two markers of meiotic germ cells^46^,^47^, (Fig. 5). In SCSKO mice, the STRA8- and SCP3-positive cells were significantly increased at 1 week of age (Fig. 5A,B, left two images), indicating that the differentiated SSCs triggered meiosis and moved toward spermatocytes. However, the number of meiotic cells was gradually reduced in the disrupted tubule of SCSKO mice beginning at 3 weeks old, which may be due to spermatocyte apoptosis (Fig. 5A,B, right images).

Gene expression profiling in 3-day-old testes demonstrated that Shp2 deletion altered the expression of numerous genes, which enriched the genes involved in SSC maintenance, such as SCF, Oct4, Etv5, and BMP4 (Fig. 5C, left table; Supplemental Fig. 4)^7^,^9^,^48^. SCF, the c-Kit ligand, is a key factor that maintains SSCs^9^. SCF expression was increased in the testes and primary SCs from the SCSKO males (Fig. 5C and unpublished data). BMP4, a growth factor belonging to TGF-β superfamily, is involved in spermatogonia differentiation^7^ and is highly expressed in SCSKO mice (Fig. 5C). In contrast, GDNF expression, a key factor for SSC self-renewal^8^, was not affected by Shp2 deficiency. These results demonstrated that Shp2 deletion promoted SSC loss by inducing excessive spermatogonia differentiation.

**Figure 6:**
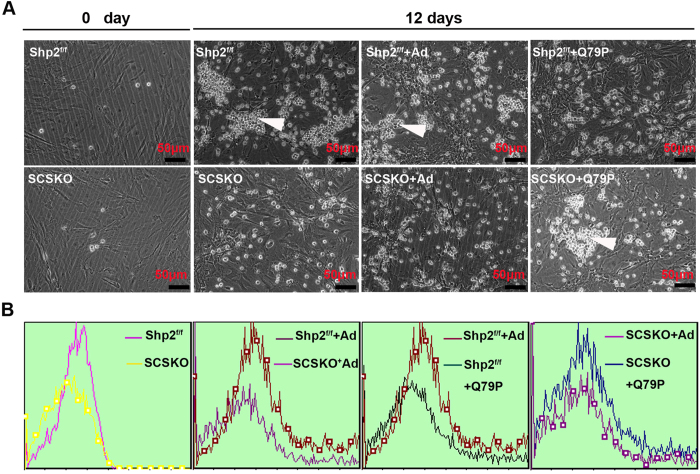
The clone formation ability of SSCs seeded on primary Sertoli cells was impaired by Shp2 deletion in Sertoli cells. (**A**) The clone formation of SSCs co-cultured with primary Sertoli cells isolated from 2-week-old mice. The arrowhead indicates the SSC clones. (**B**) Quantification of the number of stem cell clones identified by Thy (CD90) staining and flow cytometry. Ad, adenovirus; Q79P, constitutively active Shp2 mutant. The experiments were replicated at least three times, and one representative result is presented.

To further explore the effect of SC-specific deletion of Shp2 on SSC maintenance, SSCs were isolated from the wild type male testes and seeded on the primary SCs from Shp2^f/f^ or SCSKO males. Then, clone formation was assessed. As shown in Fig. 6, the SSCs seeded on the normal SCs formed clones after 12 days in culture (Fig. 6A, middle two images in the top panel). In contrast, few clones could be formed by SSCs co-cultured with the SCSKO SCs (Fig. 6A, middle two images in the bottom panel). Interestingly, the SSCs recovered their capacity to form clones when they were co-cultured with the SCSKO SCs infected with the adenovirus expressing Shp2/Q79P (Fig. 6A, right images in the bottom panel). However, SSCs lost the capacity to form clones when they were co-cultured with Shp^f/f^ SCs expressing the Shp2Q79P mutant (Fig. 6A, right images in the top panel). These results demonstrate that Shp2 is a critical protein to maintain SSCs and that the proper activity of Shp2 is necessary to maintain the balance between SSC self-renewal and differentiation. Furthermore, the germ cells of the clones were collected and labeled with Thy (CD90), a well-known marker of SSCs^49^. Then, the Thy-positive cells were analyzed by flow cytometry. Germ cells co-cultured with Shp2^f/f^ SCs displayed a strong Thy+ signal (Fig. 6B, red peak in left panel), but few Thy-positive cells were observed when the SSCs were seeded on the SCSKO SCs (Fig. 6B, yellow peak in left panel). Similarly, the restoration of Shp2 activity in the SCSKO SCs also augmented the number of Thy-positive SSCs (Fig. 6B, right first panel), and excessive Shp2 activity in wild type SCs decreased the Thy+ signal in the SSCs (Fig. 6B, right second panel). These results solidly confirmed that Shp2 is a necessary protein for SSC maintenance.

Together, these results suggested that the ablation of Shp2 in SCs disrupted the balance between SSC self-renewal and differentiation and shifted the balance toward differentiation. As a result, the pool of SSCs was depleted.

### Shp2 is required for the FSH- and testosterone-induced gene expression

**Figure 7:**
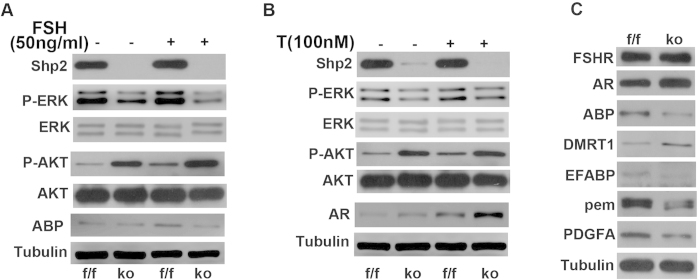
Shp2 deletion disturbed the activation of cytoplasmic signaling pathways and the expression of FSH- and testosterone-induced target genes. (**A**,**B**) The expression of a few target genes and Erk and Akt phosphorylation in primary Sertoli cells treated with FSH (**A**) or testosterone (**B**). The full-length blots are presented in Supplemental Figure S10. (**C**) The expression of FSH- or testosterone-target genes in the testes from two-week-old mice. Tubulin was used as a loading control. The full-length blots are presented in Supplemental Figure S11. The experiments were replicated at least three times, and one representative result is presented.

As a receptor-associated protein, Shp2 typically triggers the MAPK or PI3K-AKT signaling pathways to mediate cytokine signaling (such as EGF, IGF-1, FGF, etc.)^22^,^23^. With the exception of the classic pathway, FSH and testosterone also function through the non-classical pathways that evoke Erk1/2 and Akt activation^19^,^20^. Thus, to further elucidate the underlying mechanisms of Shp2 regulation, Erk1/2 and Akt activity were examined in primary SCs with or without 15 minutes of FSH (50 ng/ml) and testosterone (100 nM) treatment (Fig. 7). FSH or testosterone stimulated Erk1/2 and Akt phosphorylation in Shp^f/f^ SCs (Fig. 7A,B, left third band in the top second and fourth panels). However, Shp2 deletion reduced the basal or hormone-induced Erk1/2 phosphorylation (Fig. 7A,B, left second or fourth band in the top second panel). However, Shp2 deficiency caused an opposite effect on Akt activity and increased the basal or FSH-induced Akt phosphorylation (Fig. 7A,B, left second or fourth band in the top fourth panel). In addition, Shp2 deletion also reduced the basal or FSH-induced expression of the androgen binding protein (ABP) (Fig. 7A, left second or fourth band in the top sixth panel) and enhanced the basal or testosterone-stimulated expression of the androgen receptor (AR) (Fig. 7B, left second or fourth band in the top sixth panel). Furthermore, the expression of several FSH or testosterone target genes was analyzed in SCSKO testes or primary SCs (approximately two weeks old). DMRT1 protein levels were increased, whereas PDGFa, fatty acid binding protein 5 psoriasis-associated (FABP5 or EFABP), and protein-energy malnutrition (PEM) proteins were decreased (Fig. 7C). These data demonstrated that Shp2 mediated FSH and testosterone regulation by triggering the cytoplasmic MAPK and PI3K-Akt pathways.

## Disscussion

Using a conditional knockout mouse model, our present study demonstrated that Shp2 plays a key physiological role in male reproduction and is a critical protein required for SSC maintenance and the formation of the BTB. Shp2 deletion in SCs initiated the untimely and excessive differentiation of SSCs and disturbed the assembly of the BTB. As a result, germ cells were depleted, and the mice exhibited SC-only infertility.

In Shp2 conditional knockout mice, the structure of the seminiferous tubules was impaired, and the germ cells exhibited a disordered arrangement, which indicated that the BTB may be abnormal. BTB integrity primarily depends upon the proper assembly of the tight junctions, gap junctions and adherent junctions between SCs or between SCs and germ cells^36^. Cx43 is the predominant gap junction protein, and it is also crucial for tight junction reassembly of the BTB^3^,^37^. Cx43 knockdown in SCs significantly reduced the integrity of the BTB and induced infertility in mice^3^,^37^. Claudin11 and JAMA are two fundamental tight junction proteins in the BTB^38^,^39^. Claudin11 or JAMA mutations in mice also caused defects in the BTB^38^,^39^. These junction proteins were arranged as a regular cycle between the basal and adluminal compartments at two weeks of age when the BTB was initially assembled in normal mice. However, in SCSKO mice, Cx43, claudin11 and JAMA were dispersed in the seminiferous tubules at 2, 3 and 4 weeks old (Fig. 3B and unpublished data). Furthermore, Shp2 deletion altered the expression of several junction genes, such as claudin 1, claudin 11 and TPA^40^,^42^. These results suggested that Shp2 mediates BTB formation by regulating the expression and localization of junctional complex proteins. Shp2 deletion in SCs blocked the original assembly of the BTB at the early stages of development. However, our results did not exclude the effect of Shp2 on the cyclic assembling-disassembling of the BTB and SSC maintenance in the adult testes because Shp2 was deleted in SCs in mice beginning at embryonic day 16. Previous studies also demonstrated that Shp2 deletion in whole testes or the injection of Shp2 inhibitors in adult mice destroyed the structure of the testes and caused germ cell loss^30^. In addition, using primary SCs, the lack of Shp2 was directly demonstrated to impair the cell junctions of SCs and the clonal formation of SSCs co-cultured with SCs from SCSKO mice. The restoration of Shp2 in SCSKO SCs rescued the cell junctions of SCs and the SSC clones.

The integrated structure of the testis is the basis for normal spermatogenesis, and an abnormal BTB could cause germ cell loss^2^,^3^,^14^. In addition, germ cells depletion is also due to the disruption of the balance between SSC self-renewal and differentiation^2^,^6^. If the balance shifts to differentiation, the SSCs will be excessively differentiated and ultimately depleted^6^. SSC maintenance is entirely dependent on somatic cells^2^. SCs secrete numerous factors that are essential for SSC maintenance and spermatogenesis, such as GDNF^8^, LIF, SCF^9^, and BMP4^7^. Here, SCF signaling is important for SSC differentiation. c-Kit is the receptor for SCF and is initially expressed in late A_al_ spermatogonia at one week after birth^45^. SCF binds to c-Kit to trigger the differentiation of A_al_ into A1 spermatogonia, which is thought to be the initial step of spermatogenesis. The appearance of c-Kit is a marker of the initiation of spermatogonia differentiation^45^. In SCSKO mice, we observed a dramatic increase in c-Kit expression compared with the control males at 3 days and 1 week after birth, and SCF expression was also increased. These data further demonstrated that Shp2 deletion in SCs triggered unsuitable c-Kit/SCF signaling and subsequently induced the untimely differentiation of the spermatogonia. BMP4 is associated with spermatogonia differentiation and is expressed in SCs; its receptors, Alk3 and BMPIIR, are specifically expressed in the mitotic spermatogonia during the first week after birth. Furthermore, BMP4 also increases c-Kit expression in Kit-negative spermatogonia^7^. In our work, increased BMP4 expression was observed in SCSKO SCs. Taken together, these data revealed that the loss of the germ cells in SCSKO mice was not a secondary effect of an aberrant BTB, Shp2 deletion induced untimely spermatogonia differentiation, and the SSC pool was subsequently exhausted.

Recently, Puri *et al.* reported that Shp2 gene deletion in mouse germ cells inhibited germ cell replenishment by suppressing the proliferation of SSCs and undifferentiated spermatogonia, thus resulting in SSC loss^30^. Combined with our findings, these results suggested that Shp2 in both SCs and germ cells was a fundamental factor for SSC maintenance. Furthermore, the Shp2 inhibitor NC87877 blocked FGF- and GDNF-triggered signaling and inhibited FGF- and GDNF-dependent SSC growth *in vitro*^30^. In our studies, Shp2 deletion in SCs did not alter GDNF and FGF expression but increased SCF and BMP4 expression. These results suggested that Shp2 not only regulated the secretion of growth factors essential for SSC maintenance in SCs but also mediated signaling by these factors in germ cells.

SCs are the foundation of testis structure and function. The defects in SC development disrupt the SC maturation and impair spermatogenesis. Here, a normal population of SCs lacking Shp2 that expressed mature such as WT1, Gata1 (unpublished data), and most hormone and growth factor receptors, including FSHR, AR, IGF-1R and EGFR (unpublished data), was noted in adult mice. These results indicated that Shp2 deletion did not affect SC maturation. Therefore, we believed that the serious infertility phenotypes were derived from SC dysfunction rather than defects in SC development. Because Shp2 is a multifunctional signaling protein, its absence may impair signal transduction in SCs. In our work, Shp2 deletion in SCs abolished the FSH or testosterone regulation of Erk1/2 phosphorylation and altered the expression of their target genes, such as SCF, BMP4, ABP and AR^11^,^41^,^44^. These data demonstrated that Shp2 mediated the regulation of FSH and testosterone by the cytoplasmic MAPK and PI3K-Akt pathways. Shp2 usually triggers cytoplasmic MAPK or PI3K-AKT signaling pathways to mediate extracellular signals, such as EGF, IGF-1, and FGF, which have important roles in regulating spermatogenesis^12^,^13^,^14^. Thus, Shp2 may also be involved in the regulation of local growth factors in spermatogenesis. Furthermore, cytoplasmic signaling pathways (MAPK and PI3K-Akt) typically serve as a bridge to other signaling pathways^12^,^14^. For example, AR interacts and activates Src tyrosine kinase, which triggers the MAPK cascade^19^,^20^. Together, Shp2 plays a key role in the crosstalk and integration of a variety of signaling pathways in SCs.

Interestingly, the forced expression of Shp2Q79P (a constitutively active Shp2 mutant) also disturbed the formation of cell junctions in normal primary mouse SCs and blocked the clone formation of SSCs co-cultured with these primary SCs. Puri and Walker *et al.* (2013) also reported that Shp2Q79R overexpression in primary rat SCs impaired the BTB structure^32^. The different effects of Shp2 on cell junctions may be explained by its dual regulation of growth factor-induced signaling^22^,^23^,^24^. Shp2 can inhibit or enhance cytoplasmic MAPK and PI3K/AKT signaling in response to a single extracellular signal^22^,^23^,^24^. The dual regulation of Shp2 on cell junctions indicated that suitable Shp2 activity was necessary for junction formation in SCs or SSC maintenance.

In summary, our study demonstrated that Shp2 plays a critical role in spermatogenesis. In SCs, Shp2 orchestrated hormone- and cytokine-dependent signaling pathways to regulate the expression of target genes, which are essential for the function of SCs in SSC maintenance and formation of the BTB. Shp2 deletion in SCs impaired its function and blocked the initial stage of spermatogenesis. Our research provided a key gene for understanding the mechanism of spermatogenesis and a novel target for male infertility therapies.

## Materials and Methods

### Transgenic mouse breeding and generative ability assay

All mice were housed under standard conditions and had free access to food and water. All experimental procedures were performed in accordance with the approved guidelines of the Animal Welfare Committee of Research Organization (X200811) of Xiamen University. To generate the tissue-specific conditionally deleted Shp2 mice, Shp2^loxp/loxp^ mice^50^ (Shp2^f/f^) were bred with Amh-Cre^33^ or inhibit-α-Cre mice^34^, and the mice were genotyped by PCR using specific primers. To assay the generative ability, adult Shp2^f/f^ and SCSKO male mice from the same litter were grouped as a pair and mated with wild type female mice. On the second day, female mice with vaginal plugs were moved into another cage and observed until the pups were born. One week later, the male mice were again mated with different wild type female mice. These mating experiments were successively repeated four times. Finally, the number of litters and pups were statistically analyzed.

### Reagents and antibodies

Primary antibodies against Shp2 (sc-280), Wt1 (sc-7385), PLZF (sc-28319), Cx43 (sc-9095), UPA (sc-14019), TPA (sc-81693), ABP (sc-32891), AR (sc-816), PDGFA (sc-128), DMRT1 (sc-10485), ERK1/2 (sc-292838), Akt1/2/3 (H-136, sc-8312) and AMH (sc-6885) were obtained from Santa Cruz Biotechnology, Dallas, Texas, USA. Antibodies for phospho-Akt (Ser473, 4060), phospho-ERK1/2 (Thr202/Thr204, 4370) and PH3 (9701s) were purchased from Cell Signaling Technology, Danvers, Massachusetts, USA. Antibodies against SCF (ab64677), Stra8 (ab49405), SCP3 (ab97672), Claudin11 (ab53041), FSHR (ab35309), GDNF (ab18956) and pem (ab31922) were purchased from Abcam, Cambridge, Massachusetts, USA. Other antibodies included JAMA (36–1700, Invitrogen, Grand Island, New York, USA) and BMP4 (SAB2700755, Sigma Chemical Co., St. Louis, Missouri, USA). All chemical reagents were purchased from Sigma-Aldrich and Solarbio (Shanghai, China).

### Histology, immunohistochemistry and immunofluorescence staining

The testes were gathered and fixed in 4% paraformaldehyde (PFA) overnight. Then, the tissues were dehydrated in a graded ethanol series, cleared in a xylene solution, and then embedded in paraffin wax. The paraffin-embedded testes were sectioned serially at 5 μm thick. The sections were dewaxed, hydrated, and stained with H/E. For immunohistochemistry, the sections were microwaved in the antigen unmasking solution, incubated in methanol/hydrogen peroxide, blocked with bovine serum albumin solution, and incubated overnight with primary antibodies at 4 °C. Finally, the signals were detected by incubating the sections with Alexa488-, Alexa594- (Molecular Probes) or horseradish peroxidase-conjugated secondary antibodies (Aves). The sections were counterstained with DAPI (IF). If the antigen was detected by immunohistochemical staining, a 3,3′-diaminobenzidine (DAB) staining colorimetric reagent was used. The slides were counterstained with H/E. All images were processed with Adobe Photoshop CS6.

### TUNEL assay

The testes were fixed and paraffin sectioned as described above. The tissues were then treated with 15 mg/ml proteinase K for 10 minutes at 37 °C and stained using the *In Situ* Cell Death Detection Kit (Roche, Basel, Switzerland) according to the manufacturer’s protocol. Finally, the sections were incubated with an anti-GFP antibody and counterstained with DAPI.

### Isolation of primary Sertoli cells

The testes were derived from 10- or 14-day-old mice and quickly washed with sterile phosphate-buffered saline (PBS). Then, the cells were isolated in sterile environment. Briefly, the testes were cut into ~1 mm^3^ pieces and digested with 1 mg/ml trypsin (Sigma) for 20 minutes at 37 °C. The mixture was centrifuged at 800 g for 5 min at room temperature, and the pellet was resuspended in 40 ml of a solution containing 1 M glycine, 2 mM EDTA, 0.01% soybean trypsin inhibitor (Sigma), and 0.8 mg DNase I. After incubation at room temperature for 10 min, the mixtures were centrifuged at 800 g for 5 min at room temperature. The seminiferous tubule pellets were further digested with 1 mg/ml collagenase I, 0.8 mg/ml DNase I and 1 mg/ml hyaluronidase type III (Sigma) for 20 minutes at 37 °C with periodic agitation. Then, mixtures of SCs and germ cells were obtained from this final digestion. The dispersed cells were plated onto culture dishes in F12/Dulbecco’s modified Eagle’s medium (DMEM) containing 10% fetal bovine serum and incubated at 37 °C and 5% CO_2_. After 6 h, the medium was changed, and the detached germ cells were removed. Finally, purified SCs were obtained.

### Western blot

For Western blot analysis, testes or SC proteins were extracted and denatured for separation by sodium dodecyl sulfate polyacrylamide gel electrophoresis (SDS-PAGE). Approximately 40 mg of protein lysate from each sample was loaded. Proteins on the nylon membrane were incubated with primary antibodies at 4 °C overnight and were incubated with horseradish peroxidase-conjugated secondary antibodies at 37 °C for 2 hours. The results were visualized with enhanced chemiluminescence.

### RNA extraction, gene microarray and real-time PCR

Total RNA was isolated from testes tissues or cells using a TRIzol solution (Invitrogen). For the microarray, the RNA was labeled and hybridized to Affymetrix DNA chips. One microgram of total RNA from each sample was reverse-transcribed to cDNA in a 20 μl reaction volume. The real-time PCR was performed using an ABI7500 PCR system, and the mRNA expression level was normalized to GAPDH mRNA and analyzed using the comparative cycle threshold method. The primers employed in these experiments are listed in Supplemental Fig. 5E.

### Biotin tracer experiment

The permeability of the BTB was assessed using a biotin tracer EZ-Link Sulfo-NHS-LC-Biotin (Pierce Chemical Co., Grand Island, New York, USA). Twenty μl of a fresh PBS solution containing 10 mg/ml biotin and 1 mM CaCl_2_ was injected into the interstitium of the testes in 2-week-old mice. The animals were euthanized 2 hours later, and the testes were immediately removed and embedded in OCT. Fluorescence images of the cryosections were captured with a confocal microscope.

### Transepithelial resistance (TER) assays of tight junctions

SCs isolated from 14-day-old mice were plated onto bicameral units (Millipore Corp, Bedford, MA,USA) coated with Matrigel (BD Bioscience, Franklin Lakes, NJ,USA) at a density of 1.5 × 10^6^ cells in DMEM:F12 1:1 medium containing 10% fetal bovine serum (FBS). Two days after the initial culture, the cells were infected with AdSHP2 Q79P or AdGFP viruses for 9 hours. The quality of the tight junctions was assessed by the TER Assay. Each day, the electrical resistance across the SC monolayer was measured with a Millicell electrical resistance system (EMB Millipore Corp, Billerica, MA,USA).

### Enrichment, culture and flow cytometry of germ cells

Normal SSCs were isolated from ICR male pups (8–10 days old). SSC isolation, and purification and culture were performed in sterile conditions, according to the descriptions in previous studies. Briefly, the testes tissues were first digested with 1 mg/ml collagenase (type IV, Sigma) for 15 min. Then, the mixtures were centrifuged, and the pellet was digested again with 0.25% trypsin/1 mM EDTA for 10 min. After centrifuging, the cell pellets were suspended in PBS containing 1% FBS.

Then, the SSCs were enriched by magnetic activated cell sorting (MACS). The dissociated testis cell suspension was incubated with micro beads and 10 μl of anti-Thy-1 antibody (30-H12; Miltenyi Biotec) for 20 min at 4 °C. After rinsing with PBS containing 0.5% bovine serum albumin (BSA) (Sigma), the Thy-1+ cells were selected by passing through an LS separation column in a magnetic field. The testis feeder cells (Sertoli cells) were separated as described above, plated on laminin-coated dishes and treated with mitomycin C (MMC).

Enriched SSCs were seeded on the feeder cells (6–10 × 10^4^ cells/cm^2^) in DMEM/F12 containing 1× non-essential amino acids (Invitrogen), 0.1 mM 2-mercaptoethanol, 1x glutamine (Gibco), 10^3 ^U/ml human recombinant leukemia inhibitory factor (LIF), 20 ng/ml mouse EGF, 10 ng/ml human basic FGF (Invitrogen), 10 ng/ml recombinant rat GDNF and 1% fetal calf serum for 7 to 15 days. The culture medium was maintained at 37 °C and changed every two days.

To assay the maintenance of SSCs co-cultured with SCs, the cells were detached, dispersed into single cells and incubated with anti-Thy-1 antibody-conjugated PE at 4 °C for 20 min. Then, Thy-positive cells (SSCs) were measured by a FACSCalibur flow cytometer (Becton Dickinson, Mountain View, CA, USA) using the CellQuest software.

### Statistics

All statistical analyses in this study were performed using Student’s *t*-test. The statistical significance was defined as P < 0.05.

## Additional Information

**How to cite this article**: Hu, X. *et al.* Deletion of the tyrosine phosphatase Shp2 in Sertoli cells causes infertility in mice. *Sci. Rep.* **5**, 12982; doi: 10.1038/srep12982 (2015).

## Supplementary Material

Supplemental files

## Figures and Tables

**Table 1:** The generative ability of transgenic male mice was evaluated by four successive matings with wild type female mice.

Genotype (♂)	Number of litters	Total number of pups	Average pups per litter
Shp2 f/f (n = 10)	37	271	7 ± 29
SCSKO (n = 10)	0	0	0
