# Supplementary material for: Deletion of the tyrosine phosphatase Shp2 in Sertoli cells causes infertility in mice
Source: Sci Rep. 2015 Aug 12;5:12982. doi: 10.1038/srep12982 (PMC4533007; doi:10.1038/srep12982)
Supplement: Supplemental files [file srep12982-s1.pdf]

Deletion of tyrosine phosphatase Shp2 in Sertoli cells causes infertility in mice

Xiaopeng Hu[1]*, Zhenzhou Tang[1, 2]*, Yang Li[2]*, Wensheng Liu[2], Shuang Zhang[3], Bingyan Wang[3], Yingpu Tian[2], Yinan Zhao[2], Hao Ran[3], Wenjie Liu[2], Gen-Sheng Feng[4], Martin M. Matzuk[5], Jianwei Shuai[1], Haibin Wang[3#], Zhongxian Lu[1, 2,#]

[1]School of Physics and Mechanical & Electrical Engineering, School of Pharmaceutical Sciences, Xiamen University, Xiamen, Fujian 361005, China;

[2] State Key Laboratory of Cellular Stress Biology, School of Pharmaceutical Sciences, Xiamen University, Xiamen, Fujian 361005, China;

[3]State Key Laboratory of Reproductive Biology, Institute of Zoology, Chinese Academy of Sciences, Beijing 100101, China;

[4]Department of Pathology, and Division of Biological Sciences, University of California San Diego, La Jolla, CA 92093, USA

[5]Department of Pathology and Immunology, Baylor College of Medicine, Houston, Texas, TX 77030, USA

*These authors have equal contribution.

#Corresponding author: Zhongxian Lu, zhongxian@xmu.edu.cn; Haibin Wang, hbwang@ioz.ac.cn

Figure S1

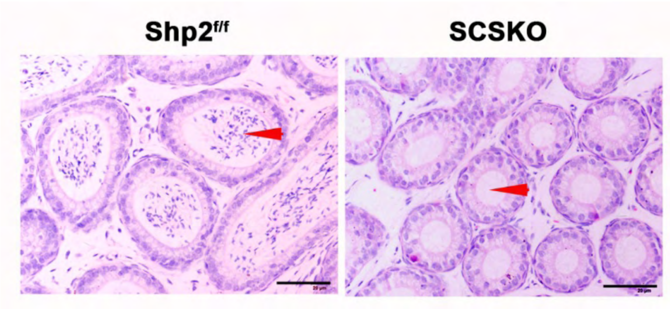

Fig S1. Mature sperms were not found in epididymis tubules of SCSKO mice. Histological Structure of epididymis was stained with H.E at 8 weeks old. Arrowheads indicated mature sperms in epididymis tubules.

Figure S2

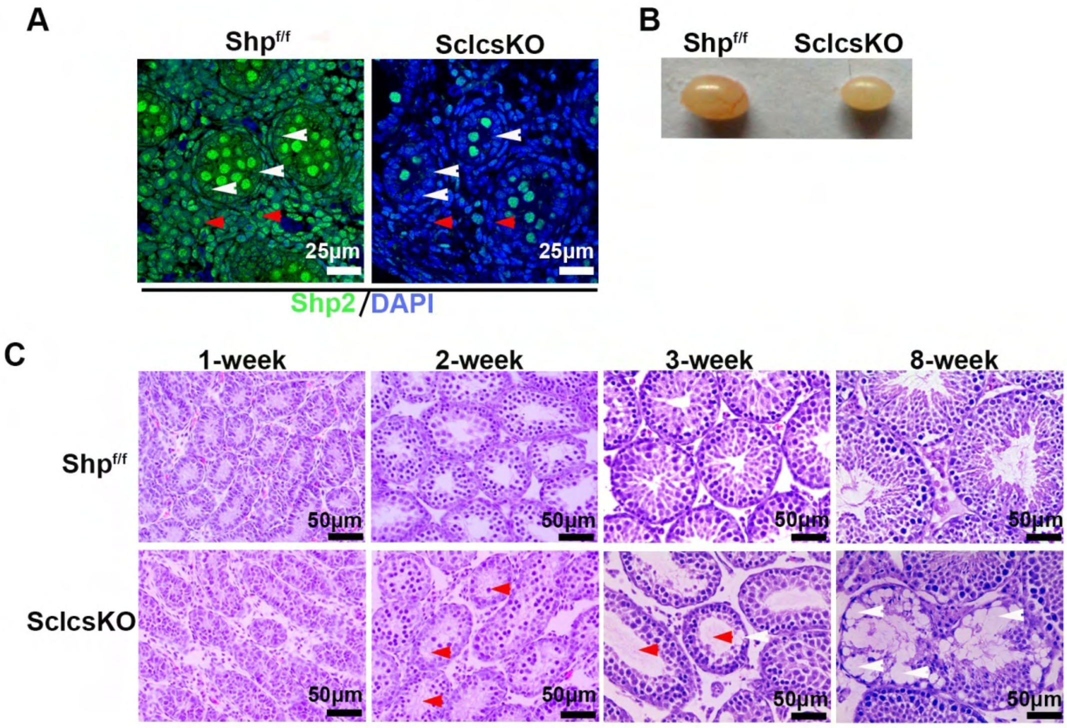

Fig S2. The development of testes was blocked and spermatogenesis was broken by the ablation of Shp2 both in Sertoli cells and Leydig cells with inhibit-α cre.. (A) Expression of Shp2 was detected by immunofluorescence staining in seminiferous tubules from Shp2$^{f/f}$ and SclcsKO male mice at E18. Arrowheads indicated Sertioli cells. Cell nucleus was stained with DAPI. (B) Stereomicroscopy images of testes from Shp2$^{f/f}$ and SclcsKO male mice at 3 weeks. (C) Testis section from postnatal 1 week, 2 weeks, 3 weeks and 8 weeks Shp2$^{f/f}$ and SCSKO mice was stained with hematoxylin and eosin (H.E). Arrowheads indicated abnormal phenotype of seminiferous epithelium.

Figure S3

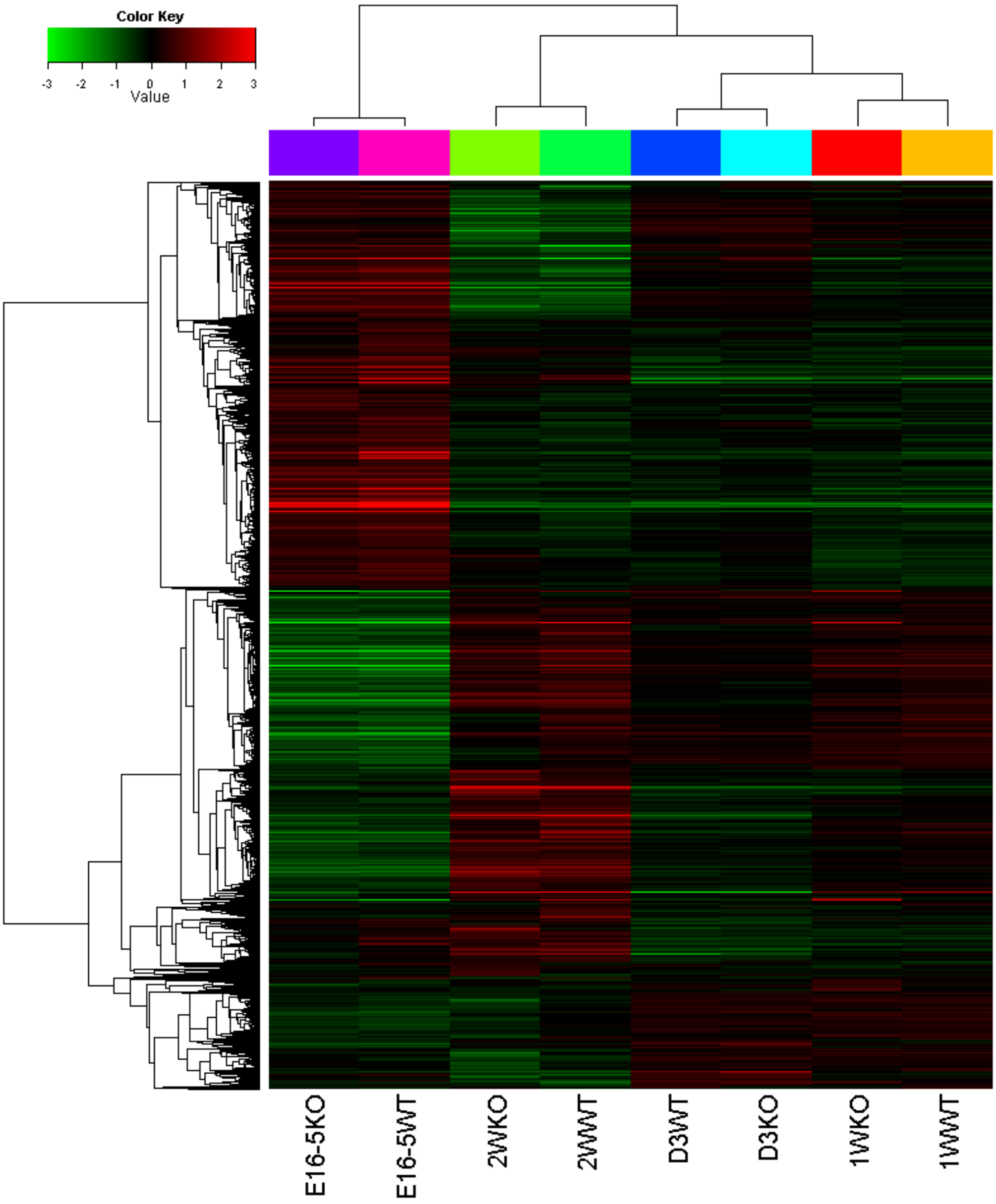

Fig S3. cluster analysis gene expression profile of testes from transgenic mice at embyro 16.5 days (E16.5), postnatal 3 days (D3), 1 week (1W) and 2 weeks (2W).

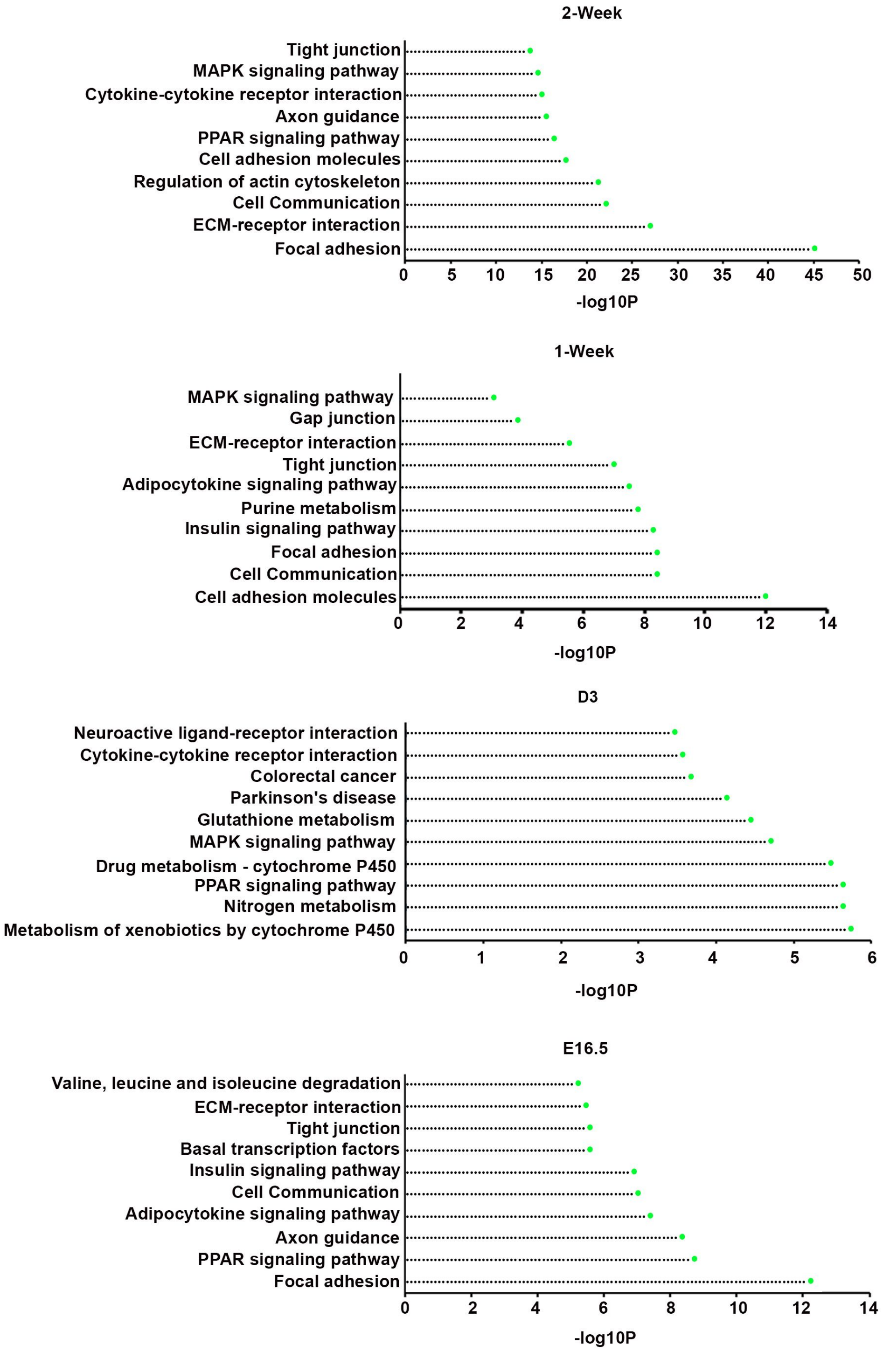

Fig S4. Enrichment of KEGG pathway in altered genes in SCSKO testes at embyro 16.5 (E16.5), postnatal 3 days (D3), 1 week (1W) and 2 weeks (2W). Altered genes is genes that expression is up- or down-regulated two fold in SCSKO compared with that in Shp2 $^{f/f}$ mice. Top ten pathways were showed in Figure. Dashed lines represent the p-values for the ten top-ranked categories of KEGG pathway associated with the gene lists. The p values are expressed as the negative logarithm (base 10). Pathway analyses were performed using DAVID (http://david.abcc.ncifcrf.gov/, Nature Protocols 2009; 4(1):44 & Nucleic Acids Res. 2009;37(1):1).

Figure S5

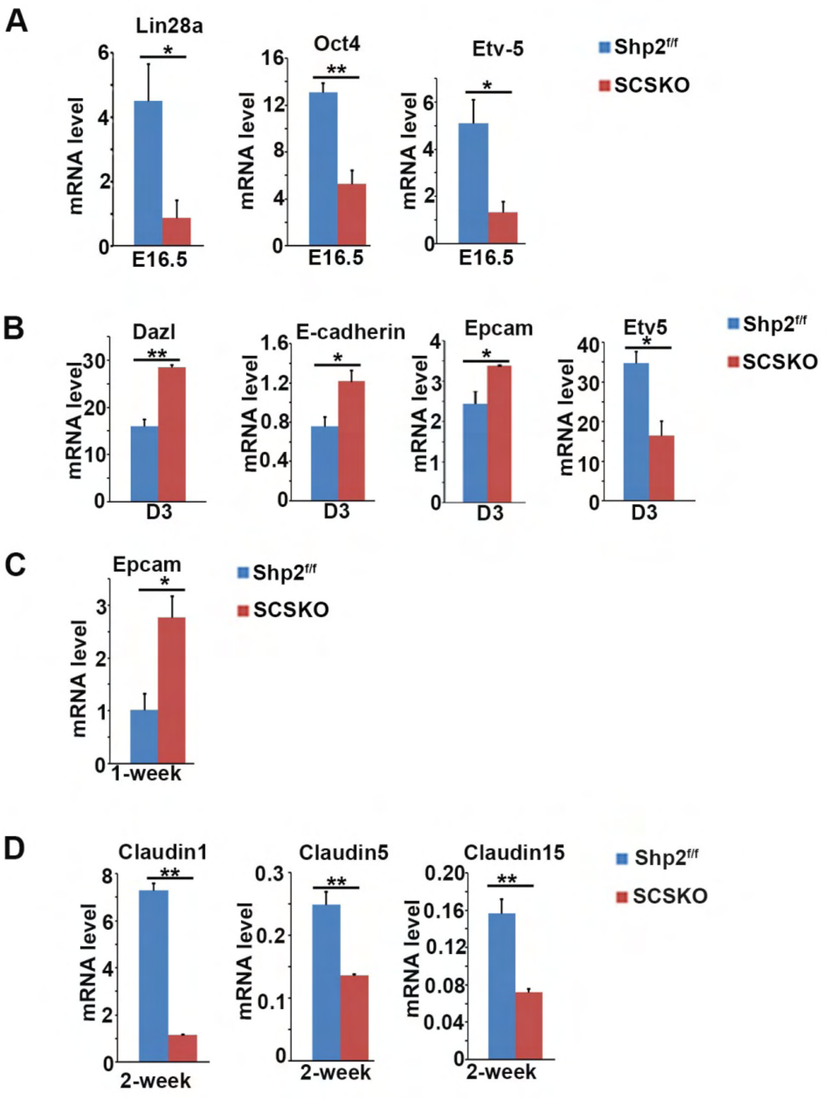

E

Table qRT-PCR primer

gene	forward Primer sequence	reverse primer sequence
Lin28a	5'CTGTGCTGGGAATGGTCTCT3'	5'AGAAAGGGCTGGGAGTGAG3'
OCT4	5'TCCCTCTGTTCCCGTCACT3'	5'TGTCTACCTCCCTTGCCTTG3'
Dazl	5'CGAAGGGCTATGGATTTGTC3'	5'GAACTGTGGTGGAGGAGGAG3'
E-cadherin	5'GGAGAACGGTGGTCAAAGAG3'	5'GCTGGCTCAAATCAAAGTCC3'
Epcam	5'CCTGAGAGTGAACGGAGAGC3'	5'GACACCACCACAATGACAGC3'
Claudin1	5'CCCGATGACCTTTCTGGAC3'	5'CAACAACAGGGTTAGCAGCA3'
Claudin5	5'GCAGAGGCACCAGAATCAAT3'	5'TCCTACCAGACACAGCACCA3'
Claudin15	5'CCAGGGATTGAGAGGGAGAG3'	5'CAGAAGACGGCAGACAAGAA3'
Etv5	5'CTGCCAGTCATCCTACAT3'	5'TACCACGCAAGTATCATCA3'
GAPDH	5'GGAGAGTGTTTCCTCGTCCC3'	5'ACTGTGCCGTTGAATTTGCC3'

Fig S5. part of altered genes in SCSKO testes were confirmed by Real-time PCR. Results were showed in panel **A** (E16.5), **B** (D3), **C** (1W) and **D** (2W). Primers in PCR were collected in table in panel **E**.

Figure S6

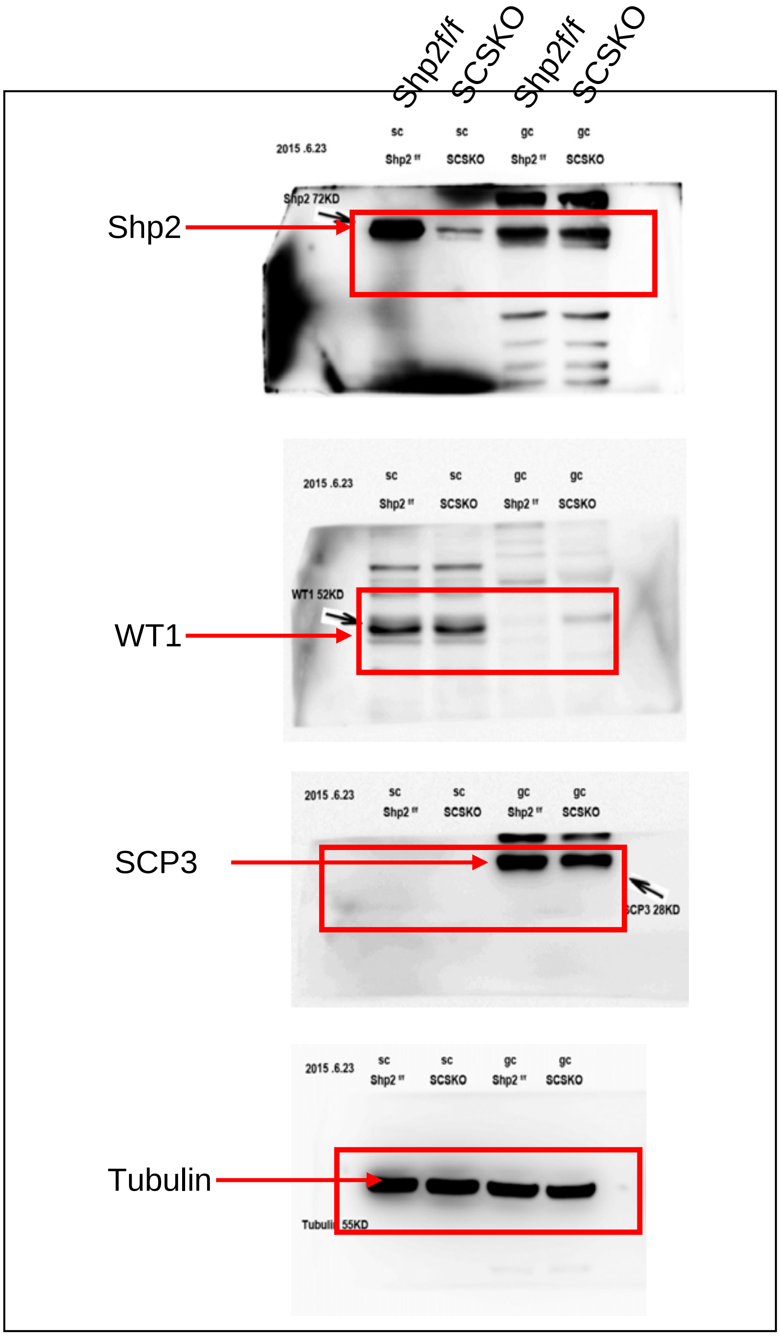

Fig S6. Full length blots of that in Figure 1B. The bands in red box were cropped. The protein level of Shp2, WT1 or FSHR was checked with western blotting in primary Sertoli cells isolated from mice at 14 days. Tubulin was a loading control. The enhanced chemiluminescent signal was detected by Image quant LAS 4000 mini.

Figure S7

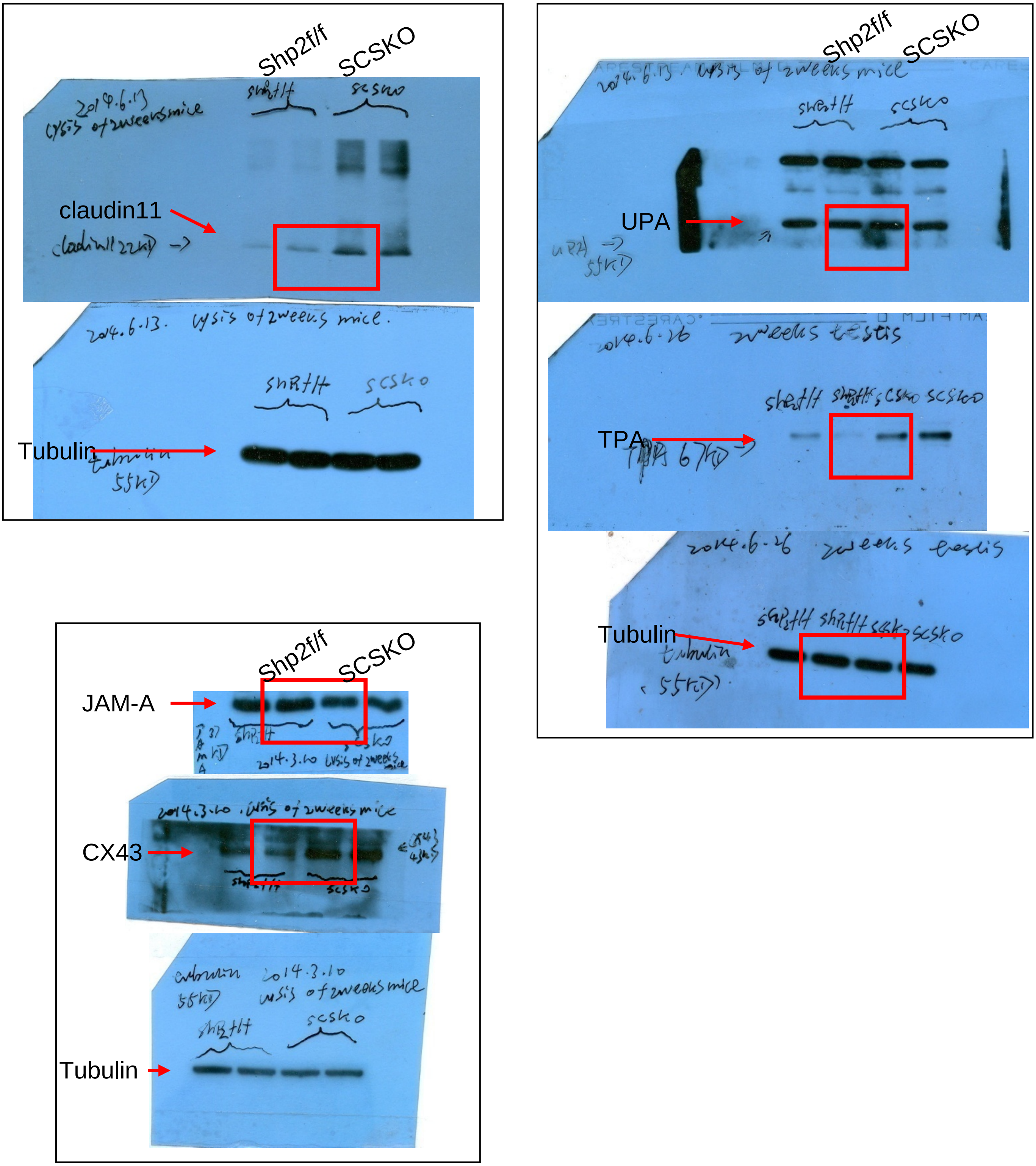

Fig S7. **Full length blots of that in Figure 3C**. The bands in red box were cropped. The protein level of part of altered genes related to BTB in microarray genes database was checked by western blotting in testes at two weeks old mice. The enhanced chemiluminescent signal was detected by X-ray film.

Figure S8

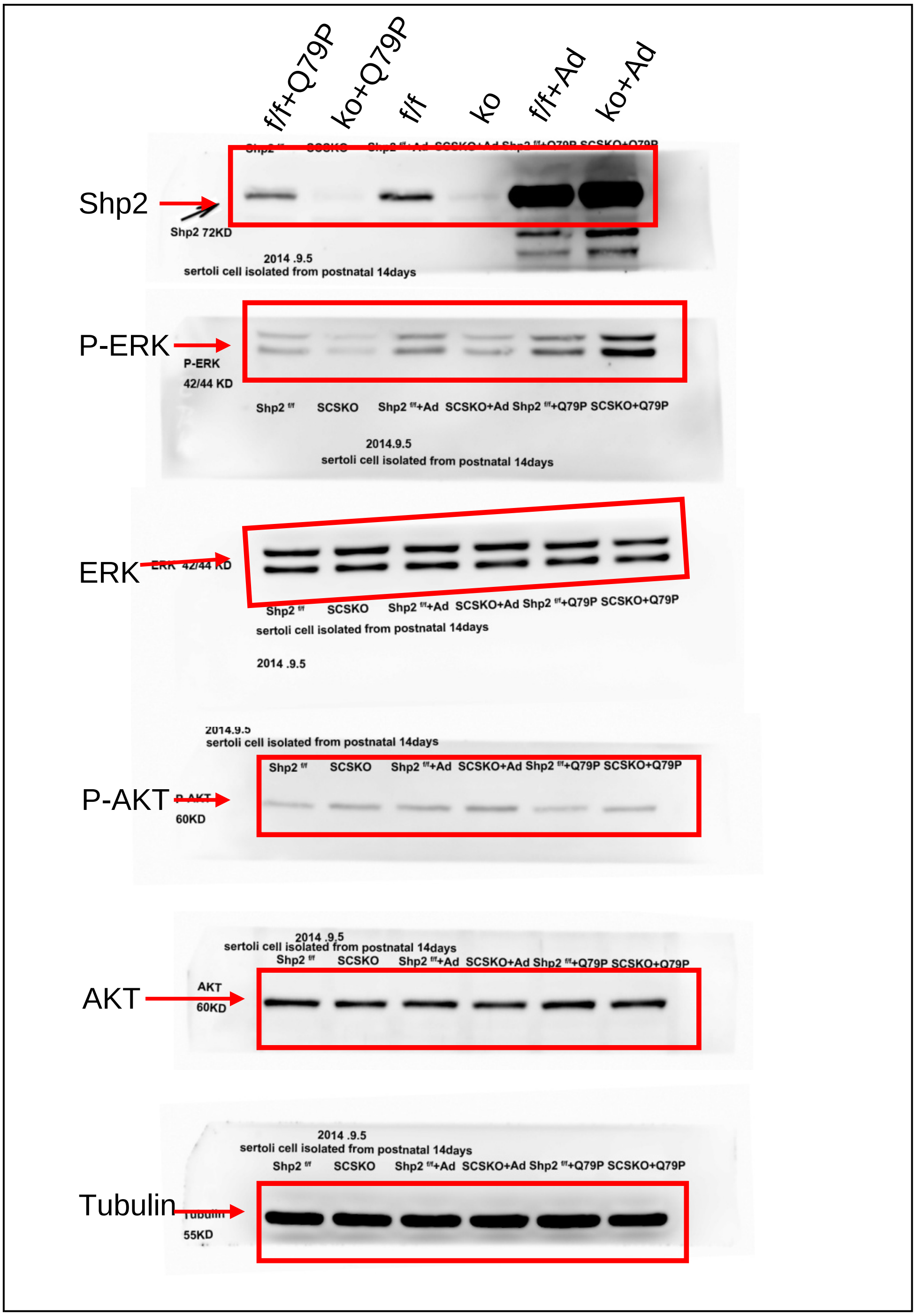

Fig S8. Full length blots of that in Figure 3E. The bands in red box were cropped. The blotting signals indicated the level of Shp2 protein and phosphorylation of Erk and Akt in primary Sertoli cells employed in Transepithelial Resistance (TER) assays. The enhanced chemiluminescent signal was detected with Image quant LAS 4000 mini.

Figure S9

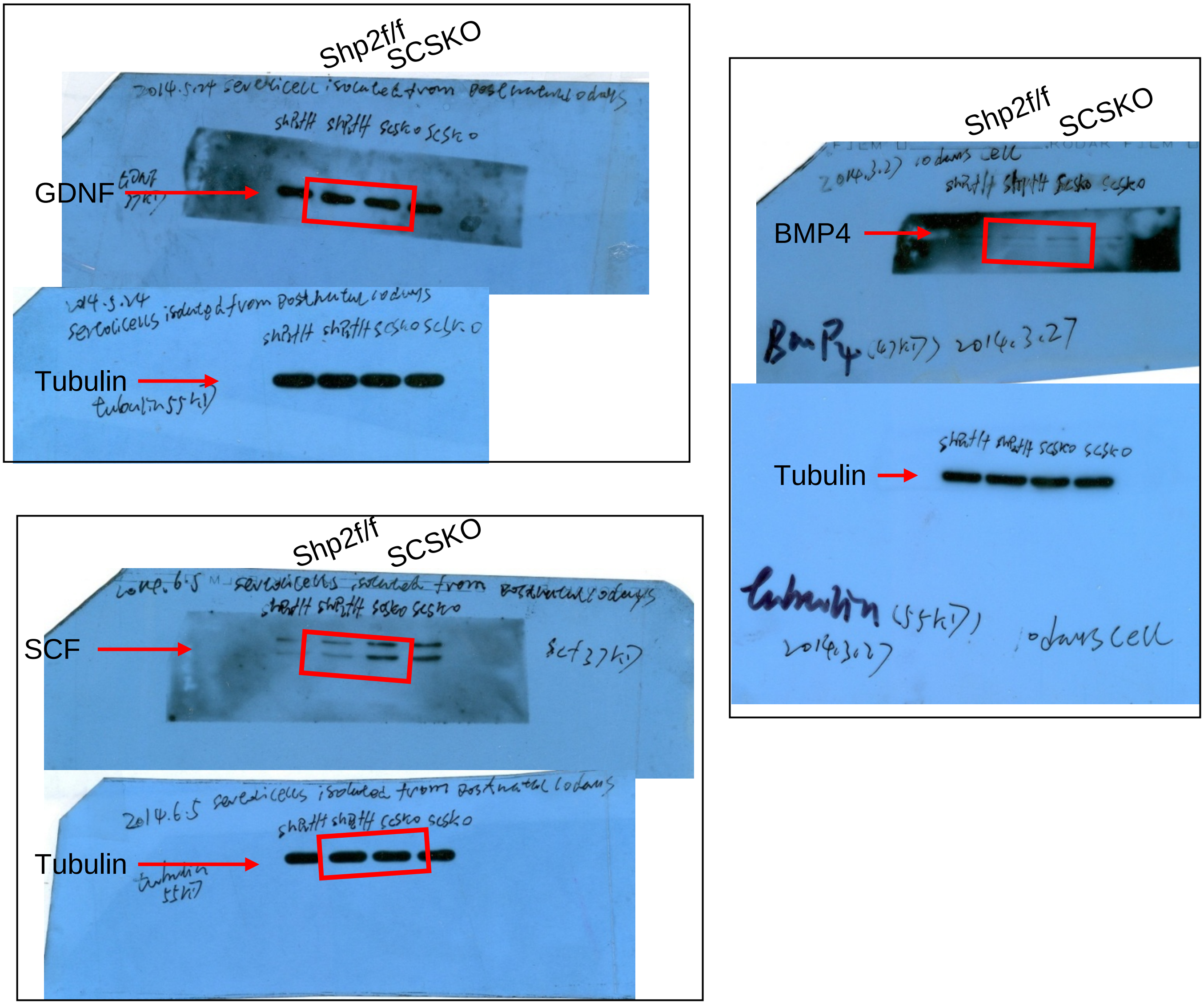

Fig S9. **Full length blots of that in Figure 5C**. The bands in red box were cropped. the protein level of part of altered genes related to the maintenance of SSCs in microarray genes database was checked by western blotting in testes at postnatal day 10. The enhanced chemiluminescent signal was detected using X-ray film.

Figure S10

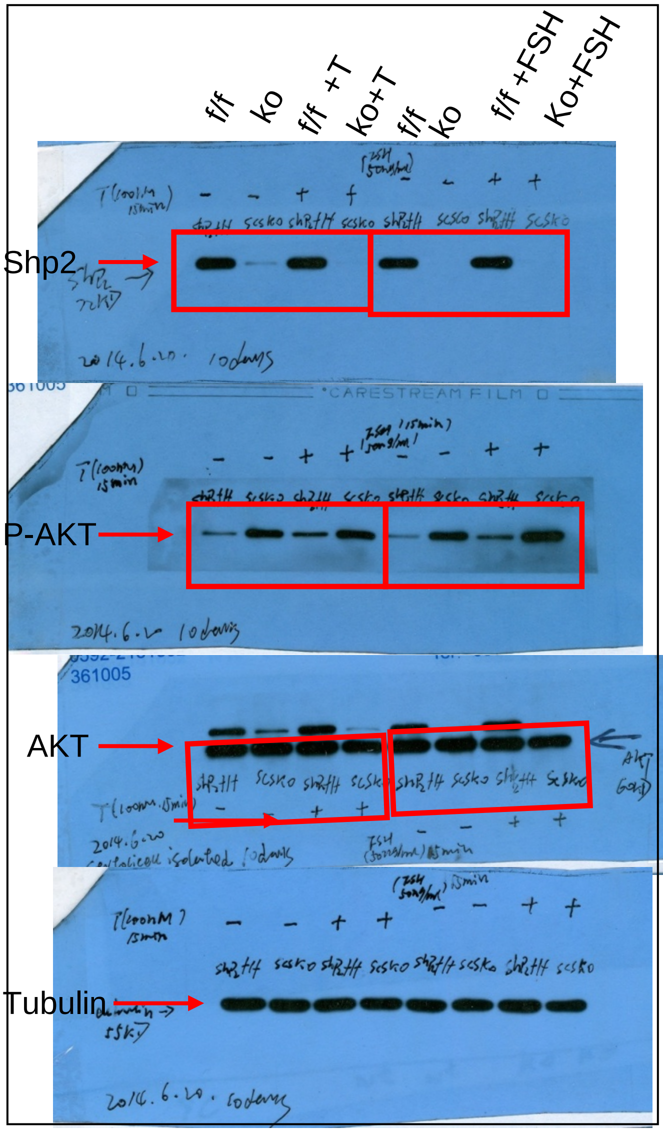

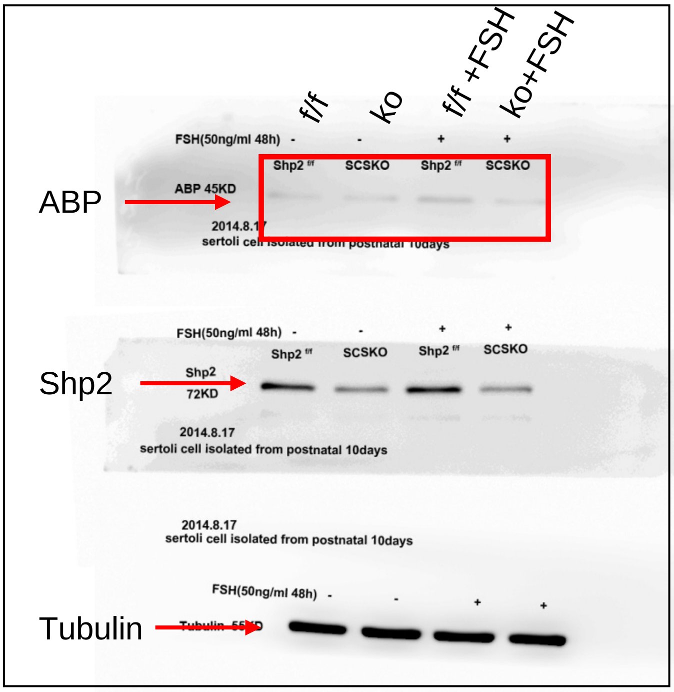

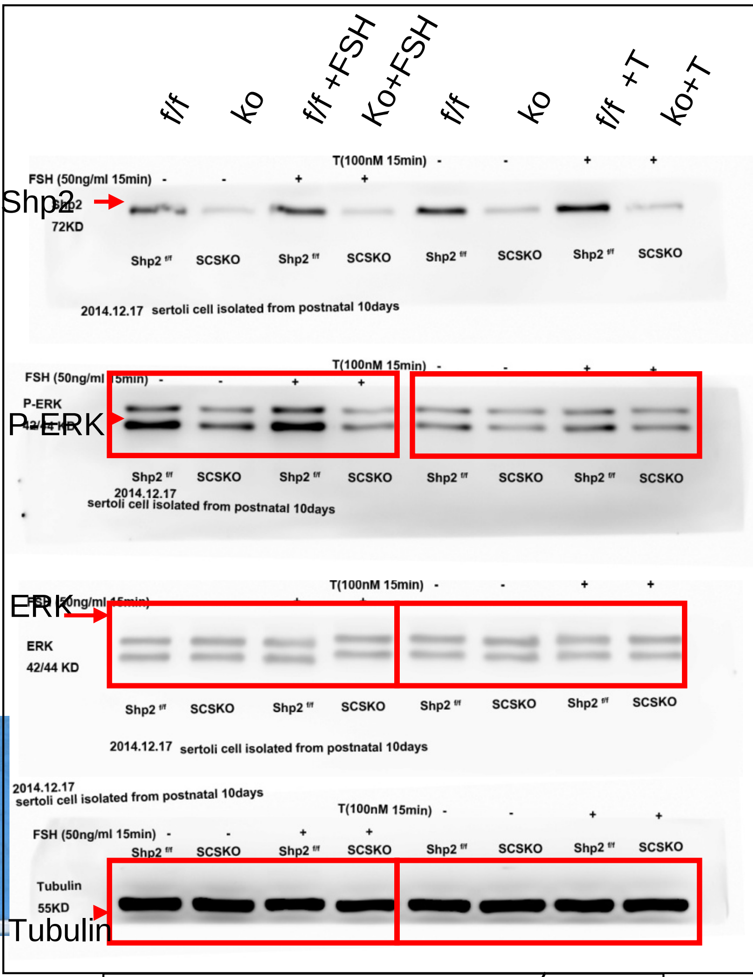

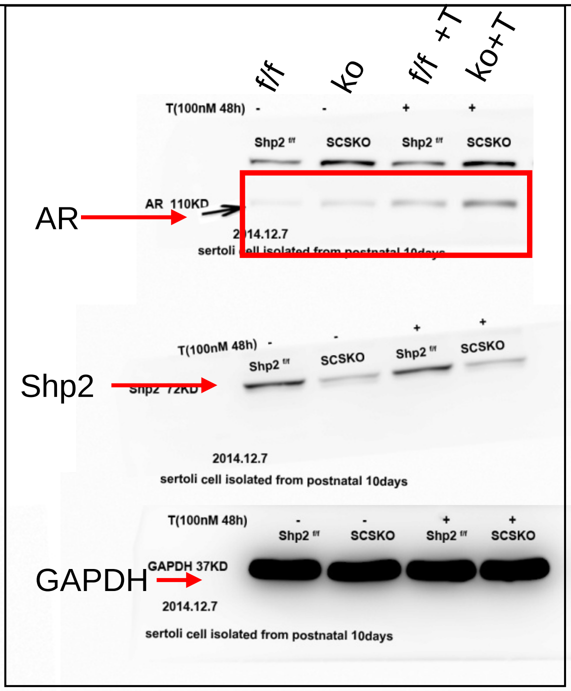

Fig S10. Full length blots of that in Figure 7A&B. The bands in red box were cropped. The enhanced chemiluminescent signal was detected by X-ray film (blue) or Image quant LAS 4000 mini (white) .

Figure S11

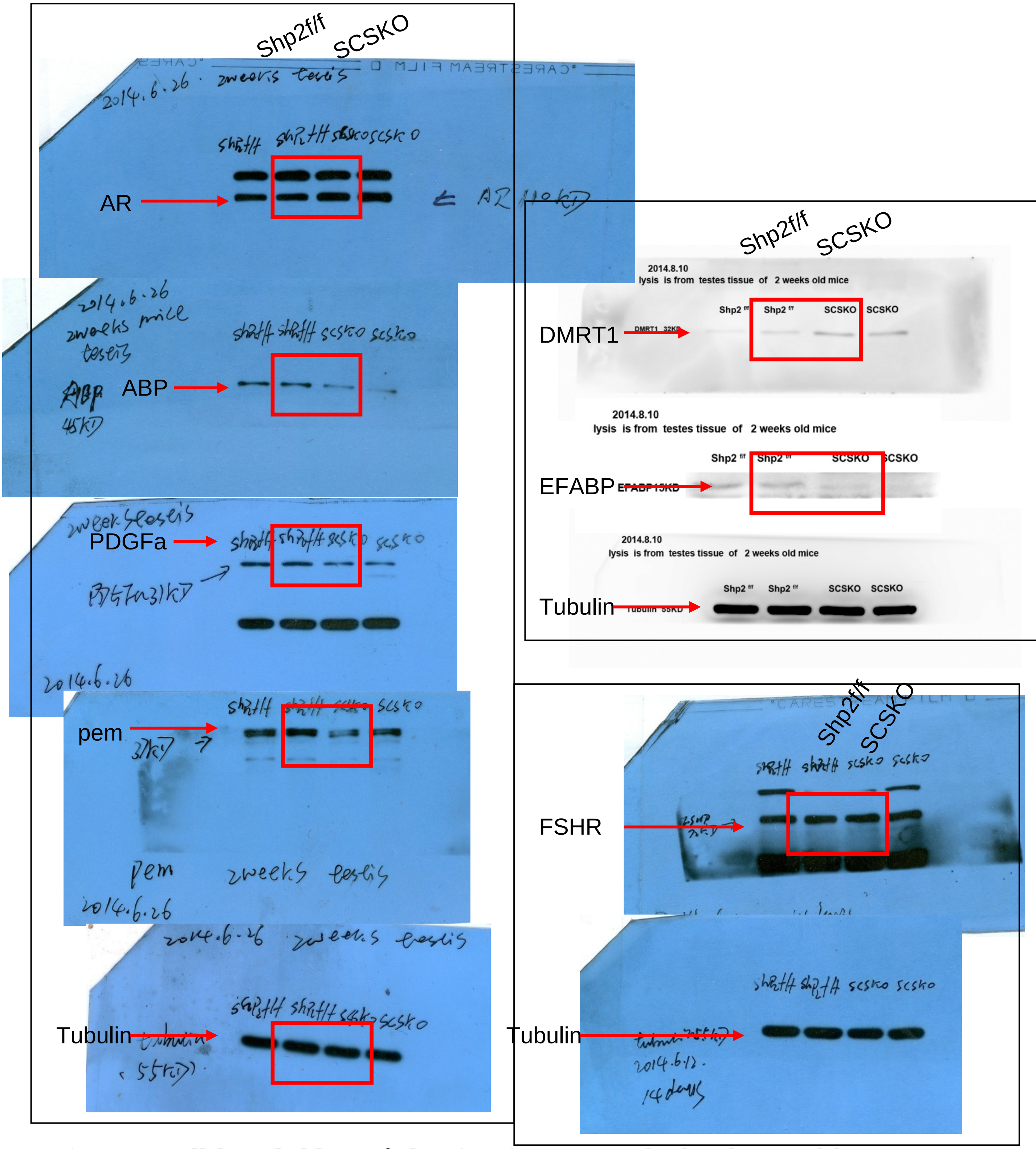

Fig S11. Full length blots of that in Figure 7C. The bands in red box were cropped. The blotting signals indicated the expression of target genes of FSH or testosterone in testes tissue. Tubulin was a loading control. The enhanced chemiluminescent signal was detected byX-ray film (blue) or Image quant LAS 4000 mini (white) .
